# Mechanical Effects of Implant Position on an Implant-Supported Removable Partial Denture Using Three-Dimensional Finite-Element Analysis

**DOI:** 10.7759/cureus.100473

**Published:** 2025-12-31

**Authors:** Kentarou Nakamura, Hirokazu Kumano, Shogo Ozawa, Jun Takebe

**Affiliations:** 1 Department of Removable Prosthodontics, School of Dentistry, Aichi Gakuin University, Nagoya, JPN

**Keywords:** dental implant, finite-element method, implant position, implant-supported removable partial denture, stress distribution

## Abstract

Purpose: This study aimed to assess the mechanical effects of different implant placement positions for removable partial dentures using the three-dimensional finite-element method.

Methods: A mandibular model with missing teeth at sites #35, #36, #45, #46, and #47 was utilized for the simulation. The basic model was designed as a removable partial denture with “Rest, Proximal plate, I-bar” clasps on teeth #34 and #44, with tooth #37 serving as an overdenture abutment featuring a magnetic attachment. For comparison, three implant-supported models were constructed, each incorporating a 10.0 mm implant placed at the edentulous posterior site #45, #46, or #47.

Results: Stress analysis revealed several mechanical effects in the implant-supported models compared with the basic model. First, the stress of the abutment tooth structures around tooth #44 decreased. Second, the stress distribution to the residual ridge mucosa also decreased. Furthermore, displacement of the denture base was markedly reduced, with smaller displacements observed at sites closer to the implant site. Among the three implant models, the model with an implant at position #46 exhibited the least displacement.

Conclusions: These findings suggest that implant placement of distal-extension removable partial dentures may reduce the mechanical load on the abutment tooth and surrounding tissues, reduce denture movement, and potentially provide an additional support area for removable partial dentures.

## Introduction

The global population continues to age at an unprecedented rate. According to the United Nations Department of Economic and Social Affairs, the number of people aged ≥65 years is projected to more than double, from 761 million in 2021 to 1.6 billion by 2050 [1]. Japan is among the fastest aging countries globally, and individuals aged ≥65 are projected to account for approximately 30% of the total population by 2025, indicating a transition to a “super-aged society” [2]. Nevertheless, due to advances in medical care and increasing health awareness, the number of remaining natural teeth among older adults has been steadily increasing. However, tooth loss remains common with advancing age, and the number of older adults using removable partial dentures continues to increase [3].

The safety and efficacy of dental implant therapy are well established. Removable partial dentures are designed utilizing implants as additional supportive elements [4]. Prosthodontic treatment is particularly suitable for elderly patients as well as those with systemic conditions that may complicate the potential of implant-supported prostheses, by allowing for the placement of a minimal number of implants, often with shorter lengths, thereby reducing surgical invasiveness [5,6]. The use of implants as supportive components can mitigate denture rotation and settling by providing additional support points between abutments. Furthermore, several reports have indicated that implant-supported removable partial dentures (ISRPDs), compared with conventional removable partial dentures, improve denture stability and support, thereby enhancing masticatory performance, patient satisfaction, and oral health-related quality of life [7-9].

However, the mechanical function of the implant support in relation to the viscoelastic properties of the mucosa and periodontal ligament remains unclear. Although several previous studies have examined the mechanical effects of implant placement of Kennedy Class II removable partial dentures, most have focused on localized outcomes, such as stress to adjacent abutment teeth and peri-implant bone. However, the global mechanical behavior of the entire denture, including the contralateral abutments, which may also be affected by implant positioning, has received limited attention [4]. Moreover, few studies have incorporated nonlinear material properties to reproduce the biomechanical behavior of the periodontal ligament and residual ridge mucosa. Therefore, the aim of the present study was to comprehensively evaluate the effects of implant position at different edentulous sites on both localized stress and overall denture behavior, including displacement and stress distribution of supporting structures, with the use of the finite-element method (FEM). In particular, nonlinear material properties were assessed to more accurately simulate the biomechanical behavior of the residual ridge mucosa and periodontal ligament. In addition, the mechanical effects of implant placement at different edentulous sites in the design of Kennedy Class II removable partial dentures were investigated using the three-dimensional FEM.

## Materials and methods

Analysis environment

Finite-element models were constructed using computer-aided engineering pre-/post-processing software (Patran 2013; 64-bit; MSC Software Corporation, Newport Beach, CA, USA). Structural analysis was performed using general-purpose, implicit nonlinear finite-element analysis software (Marc 2010; MSC Software Corporation).

Analysis model

**Figure 1:**
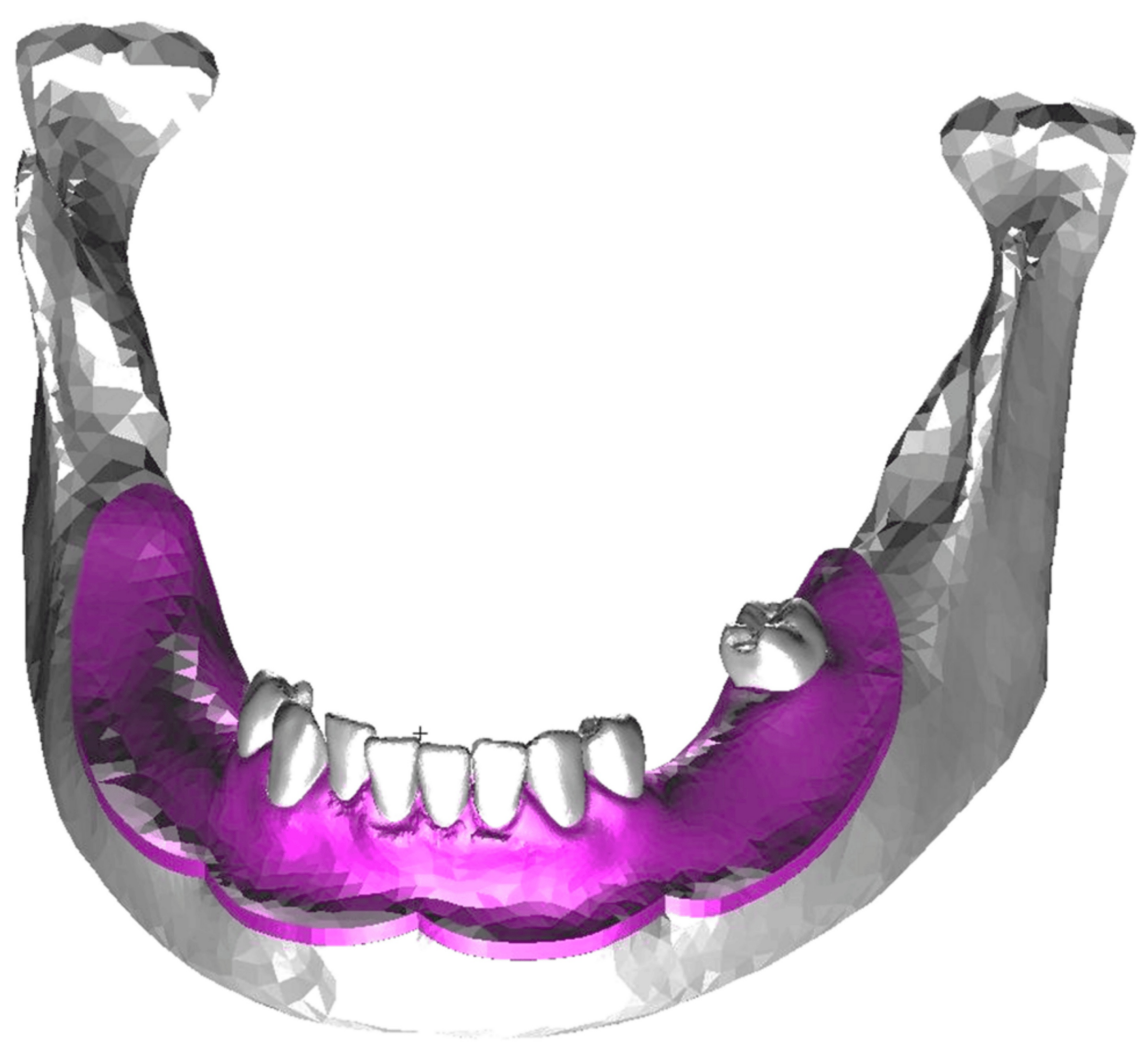
The mandibular model utilized in this study

The mandibular model created for this study is presented in Figure 1.

**Figure 2:**
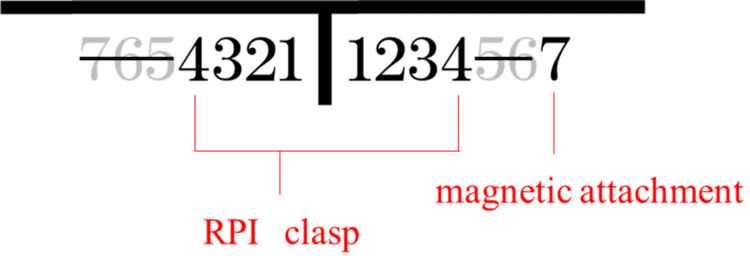
Design of the removable partial denture RPI: Rest, Proximal Plate, I-Bar

The geometry was constructed using a mandibular gypsum model (A.G.U/PD2; Nissin Dental Products Inc., Kyoto, Japan) and a skull model (P10-SB.1; Nissin Dental Products Inc.). Initially, the mandibular gypsum model was scanned using a three-dimensional scanner (7 series; Dental Wings, Inc., Montreal, Canada) to generate stereolithography (STL)-format geometry data. As the gypsum model lacked anatomical details of the contour of the mandibular bone and morphology of the tooth roots, the skull model was scanned with a computed tomography (CT) system. The CT data were subsequently processed using Mimics three-dimensional reconstruction software (version 11.0; Materialise NV, Leuven, Belgium) to generate STL data representative of the mandibular bone and root geometries. The combined STL data were then imported into Patran software to construct a complete mandibular model. Considering regional variation in residual ridge mucosal thickness, site-specific mean mucosal thickness values were assigned based on Munakata et al. [10]. The design of the removable partial denture is provided in Figure 2.

The denture consisted of a removable partial denture with a metal framework, featuring a magnetic attachment placed on tooth #37 and “Rest, Proximal plate, I-bar” clasps applied to teeth #34 and #44 as supportive components. The mandibular denture model was fabricated from a wax pattern of the keeper-attached basal coping, a wax denture, and a metal framework of the research model. The models were digitized as described above. The mandibular and denture models were combined and imported into the Patran software to generate a basic finite-element model for subsequent analysis [11,12]. The finite-element mesh was generated using tetrahedral and wedge elements, and mesh density was locally refined while maintaining element quality.

Analysis items

**Figure 3:**
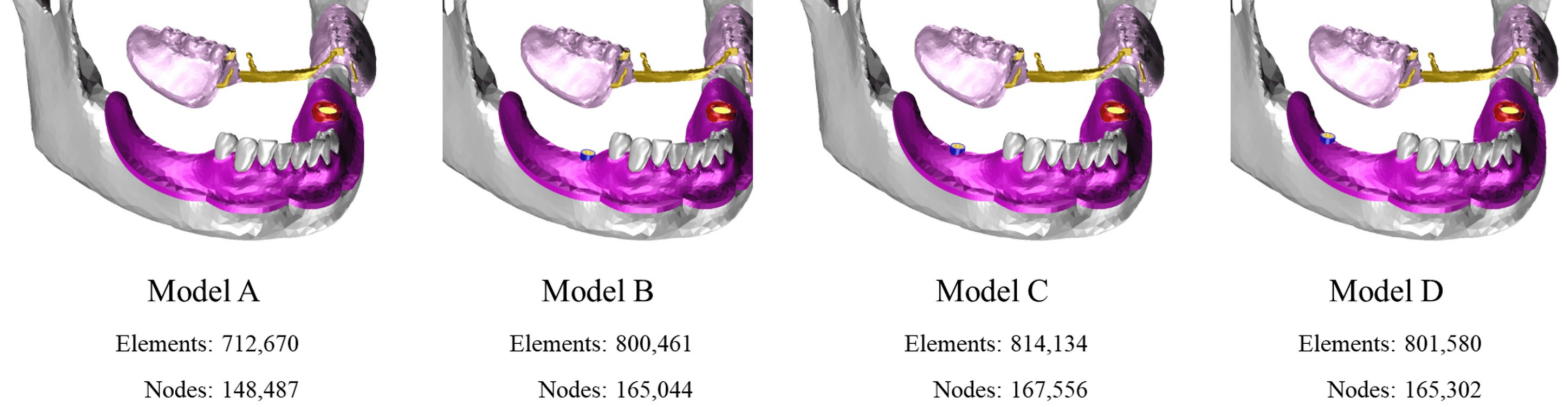
Analysis models Model A: basic model without implant support. Model B: model with an implant at #45. Model C: model with an implant at #46. Model D: model with an implant at #47

Four finite-element models were constructed for analysis: a basic model without implant support (Model A) and three implant-supported models (Models B-D), each incorporating a single 10.0-mm implant placed at one edentulous site corresponding to tooth #45, #46, or #47, respectively (Figure 3).

**Figure 4:**
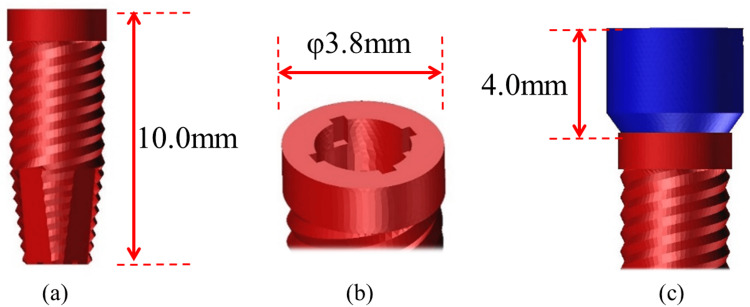
Implant body model (a) Implant body, lateral view (length 10.0 mm). (b) Implant body, occlusal view (diameter φ 3.8 mm). (c) Healing abutment, lateral view (height 4.0 mm)

A healing abutment (height, 4.0 mm) was attached to each implant and designated as a supporting area beneath the denture base (Figure 4). The implants employed in this study were modeled in reference to a commercially available dental implant (Genesio® Plus Straight; GC Corporation, Tokyo, Japan).

The mechanical properties of each component of the finite-element models are described in Table 1 [11-15].

**Table 1:** Mechanical property values

Material	Young’s modulus (MPa)	Poisson’s ratio
Mandibular bone	11,760	0.25
Enamel	41,400	0.35
Dentin	18,600	0.35
Resin	2,450	0.3
Co-Cr	200,000	0.3
Gold alloy	142,000	0.39
Titanium	104,100	0.34
Ti-6Al-4V	113,800	0.34

For the periodontal ligament and residual ridge mucosa, material nonlinearity was incorporated using a material constant conversion program, as detailed in Table 2 [11,12]. This program sequentially modifies the material constants in a piecewise manner, where the inflection points are defined based on experimentally measured in vivo load-response data at predetermined load levels. This approach enables the nonlinear mechanical behavior of the periodontal ligament and residual ridge mucosa to be approximated by updating the stiffness parameters as the applied load increases.

**Table 2:** Material constant conversion program

Tissue	Young’s modulus (MPa)	Poisson’s ratio
Periodontal ligament		
1st	0.020	0.200
2nd	0.085	0.300
3rd	1.500	0.350
4th	2.500	0.400
5th	4.000	0.490
Residual ridge mucosa		
1st	0.150	0.300
2nd	0.700	0.350
3rd	3.000	0.350
4th	3.900	0.350
5th	4.600	0.450
6th	11.000	0.470
7th	16.500	0.490

Analysis conditions

**Figure 5:**
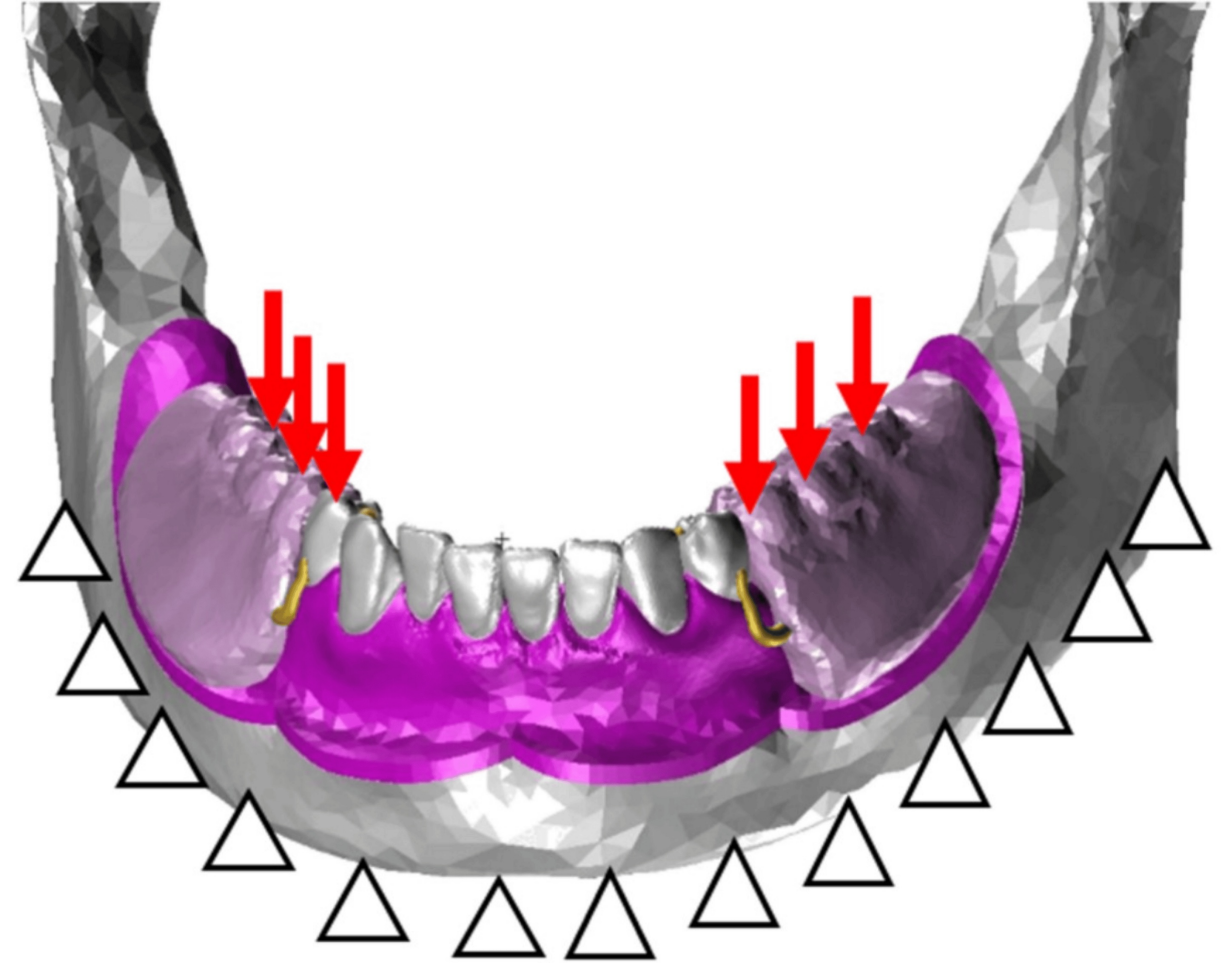
Load conditions

The loading conditions are described in Figure 5. Loads were applied at six distinct locations corresponding to the central fossae of the occlusal surfaces of teeth #35, #36, #37, #45, #46, and #47. Each load was applied perpendicularly to the occlusal plane. The magnitude of each load was 50 N, with a total load of 300 N distributed across all six sites [16]. The boundary conditions were defined by fully constraining the inferior border of the mandibular bone model in the X, Y, and Z directions [12]. Coulomb friction (coefficient of friction μ = 0.09) was applied to the areas in contact with the denture [17].

Evaluation parameters

In this study, six evaluation parameters were defined to assess the mechanical effects of the implant placement position on the denture and supporting tissues.

Displacement of the Denture

**Figure 6:**
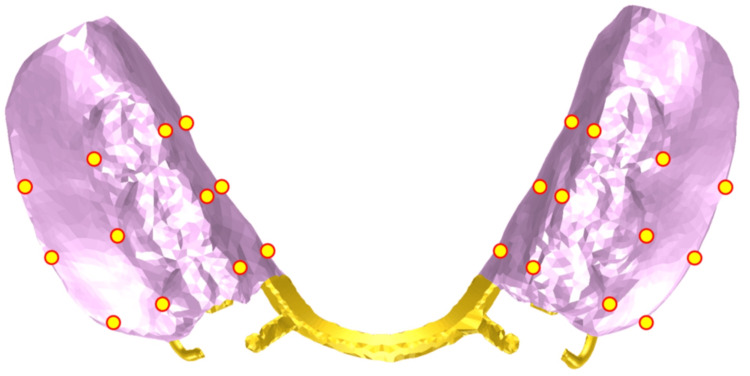
Measurement point locations for overall denture displacement analysis

To analyze the displacement behavior of the denture, multiple measurement points were positioned at the cervical and border regions of the denture base. Displacement vectors along the X-, Y-, and Z-axes at each point were calculated. The measurement point locations are illustrated in Figure 6.

Vertical Displacement of the Denture Base

**Figure 7:**
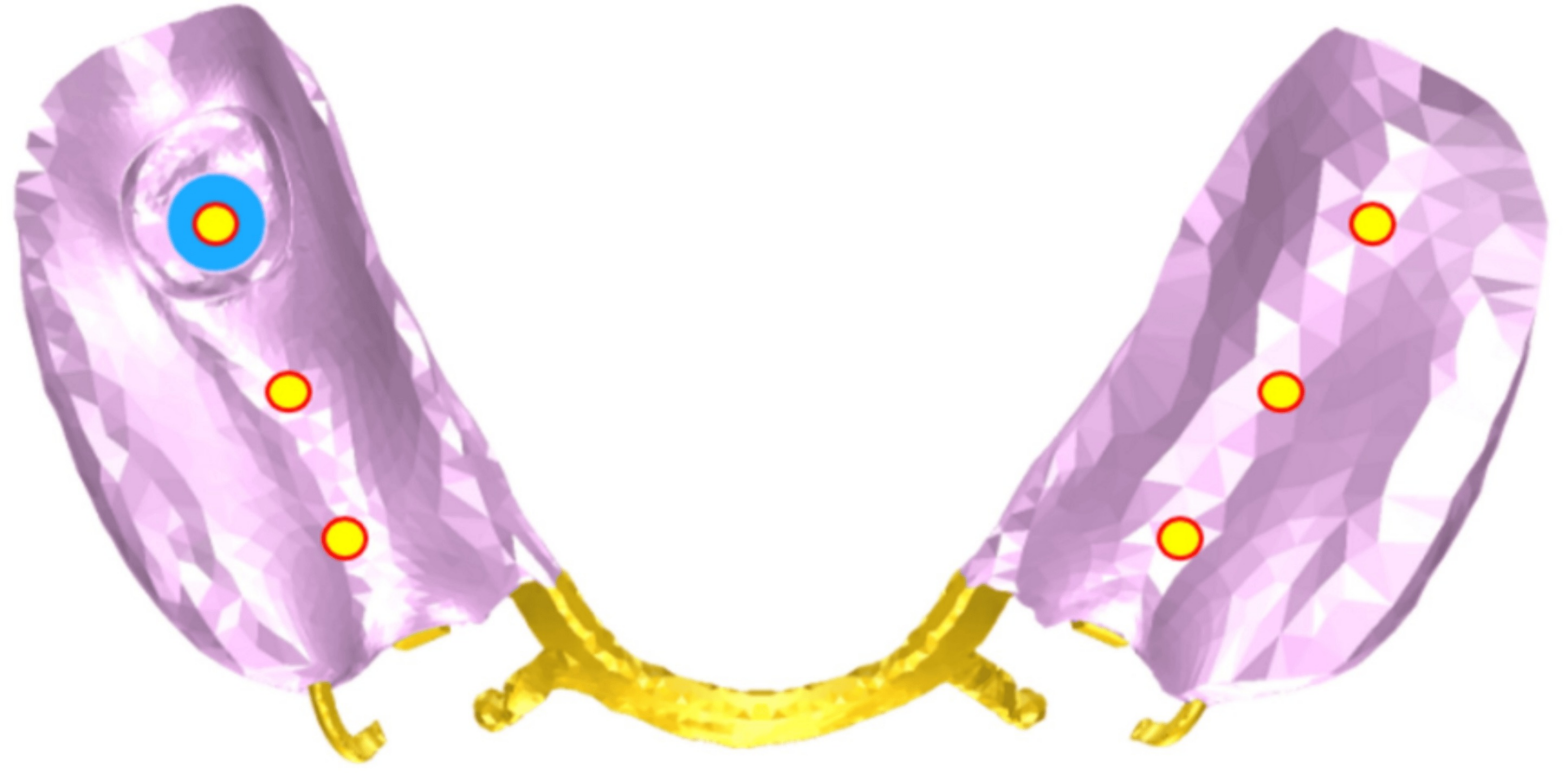
Measurement point locations for vertical displacement (settling) of the denture base

To evaluate the vertical settling behavior of the denture at the supporting sites, the measurement points were positioned at locations corresponding to teeth #35, #36, #37, #45, #46, and #47. Displacement along the Z-axis at each point was measured, as illustrated in Figure 7.

Displacement of the Abutment Teeth

**Figure 8:**
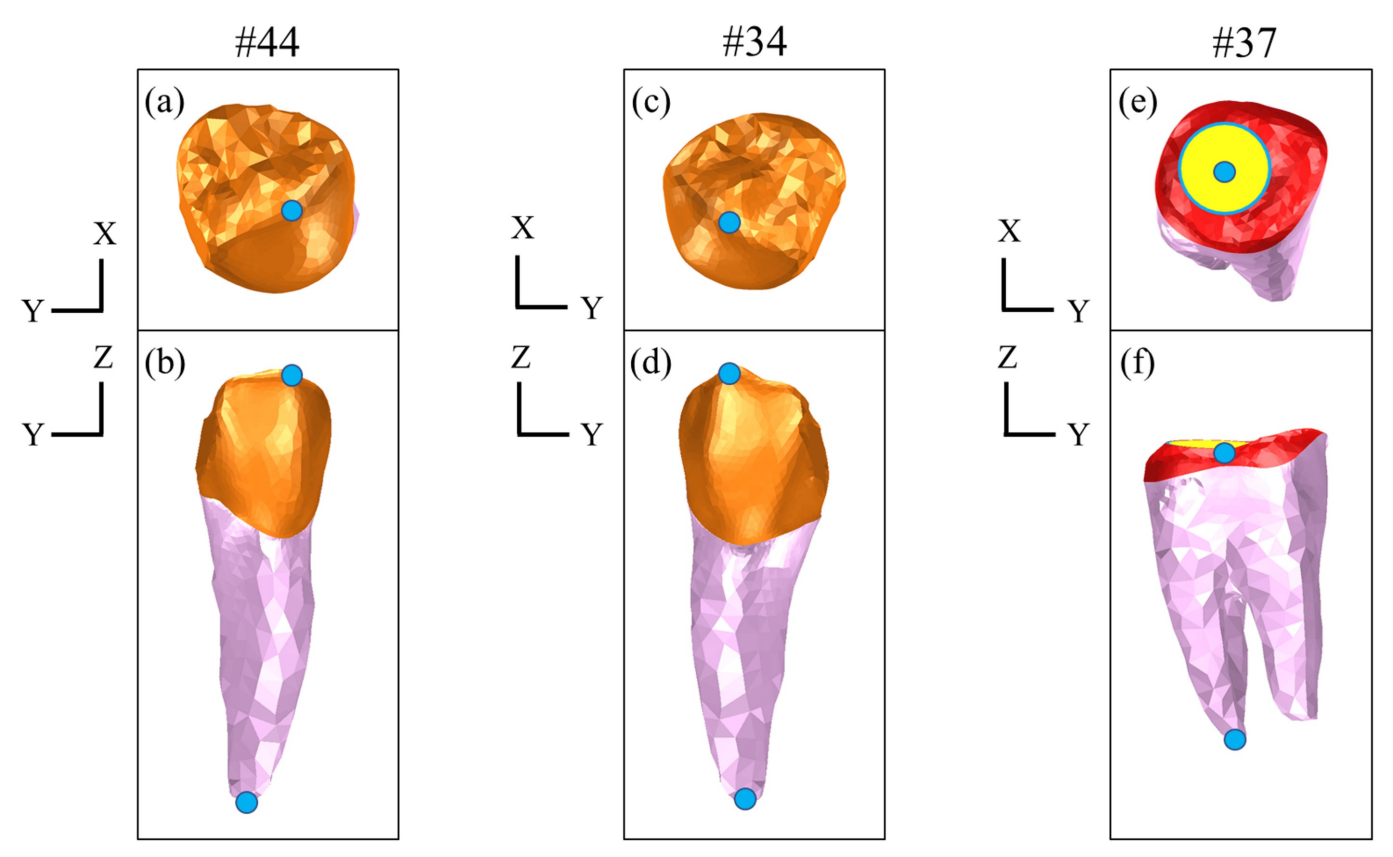
Measurement point locations of abutment teeth for displacement analysis (a) Tooth #44, occlusal view: measurement point at the crown (blue marker). (b) Tooth #44, buccal view: measurement points at the crown and root apex (blue markers). (c) Tooth #34, occlusal view: measurement point at the crown (blue marker). (d) Tooth #34, buccal view: measurement points at the crown and root apex (blue markers). (e) Tooth #37, occlusal view: measurement point at the crown (blue marker). (f) Tooth #37, buccal view: measurement points at the crown and root apex (blue markers)

To assess the mechanical effects on the abutment teeth, displacement vectors (X-, Y-, and Z-components) were calculated at both the crown and root apex of teeth #34, #44, and #37, which directly engaged with the denture framework. The displacement direction and magnitude were compared using both occlusal and buccal views. The measurement point locations are illustrated in Figure 8.

Stress Distribution of the Residual Ridge Mucosa

Von Mises equivalent stress was employed to evaluate the stress distribution of the residual ridge mucosa in contact with the denture base.

Stress Distribution of the Alveolar Bone Around the Abutment Teeth

Von Mises equivalent stress was employed to evaluate the stress distribution of the alveolar bone surrounding the abutment teeth (#34, #44, and #37).

Stress Distribution of the Peri-Implant Bone

In the implant-supported models, the minimum principal stress distribution of the peri-implant bone was assessed.

## Results

Displacement of the denture

**Figure 9:**
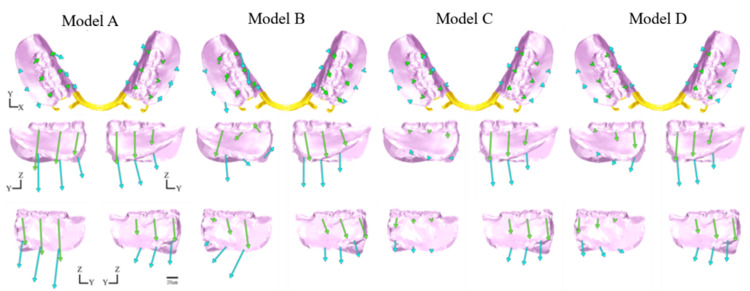
Displacement of the denture Displacement vectors of the denture in each model. Analysis models. Model A: basic model without implant support. Model B: implant placed at #45. Model C: implant placed at #46. Model D: implant placed at #47. The top, middle, and bottom rows show the occlusal, buccal, and lingual views, respectively

The overall displacement behavior of the denture is illustrated in Figure 9. The displacement directions of the denture in each model were compared based on the implant placement positions. The evaluation parameters included the magnitude and directional components (X-, Y-, and Z-axes) of the displacement vectors at representative points on the denture.

Occlusal View

From the occlusal view, the displacement behavior along the XY plane varied depending on the implant location. Compared to Model A, the lateral movement of the denture in Model B tended to increase. In contrast, lateral movement was suppressed, and overall displacement was reduced in Models C and D. Notably, Model D exhibited greater suppression of denture displacement than Model C.

Lateral View

From the lateral view, differences in the displacement patterns along the YZ plane were dependent on the implant position. In particular, the Z-axis displacement, reflecting denture settling or lifting, served as an important indicator, with notable differences among the models. In Model B, the denture exhibited posterior rotational movement, accompanied by large anteroposterior displacement. In contrast, Model C exhibited the least displacement along the Z-axis, indicating minimal settling, and displacement along the Y-axis was minimal compared with the other models. Thus, Model C had the most stable configuration from the lateral view. Displacement along the Z-axis was greater for Model D than Model C, with a tendency toward increased settling mesially from the implant site. Overall, Model D exhibited anterior rotational movement of the denture.

Displacement of the denture base

**Figure 10:**
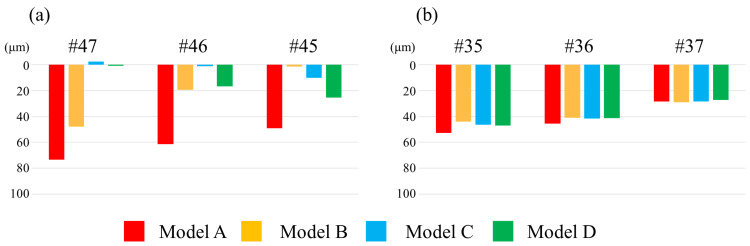
Degree of displacement of the denture base (a) At sites #47, #46, and #45. (b) At sites #35, #36, and #37

Figure 10 illustrates the vertical displacement of the denture base. In the regions corresponding to sites #35, #36, and #37, no marked differences in displacement were observed among the models, and the displacement tended to increase toward the mesial side. In contrast, at the regions corresponding to sites #45, #46, and #47, the displacement of all implant-supported models was decreased compared to Model A. For each implant-supported model, the closer the measurement point approached the implant site, the smaller the vertical displacement became. In Model B, compared to Model A, vertical displacement was reduced at sites #45, #46, and #47. The least displacement occurred at #45, with displacement increasing toward sites #46 and #47. In Model C, compared to Model A, displacement was reduced at sites #45, #46, and #47. The least displacement occurred at site #46, with displacement increasing toward the mesial side. In Model D, compared to Model A, displacement was reduced at sites #45, #46, and #47. The least displacement occurred at #47, with displacement increasing toward sites #46 and #45.

Displacement of the abutment teeth

**Figure 11:**
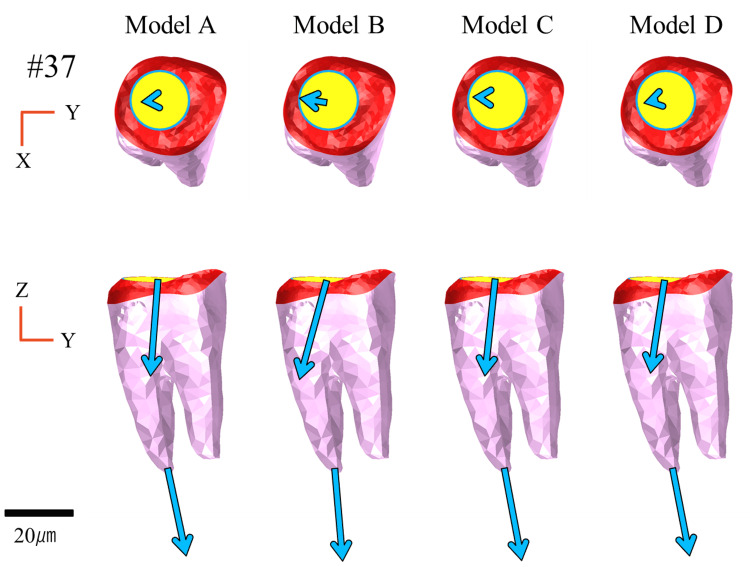
Displacement of abutment teeth (#37) Analysis models. Model A: basic model without implant support. Model B: implant placed at #45. Model C: implant placed at #46. Model D: implant placed at #47. The top and bottom rows show the occlusal and buccal views, respectively

**Figure 12:**
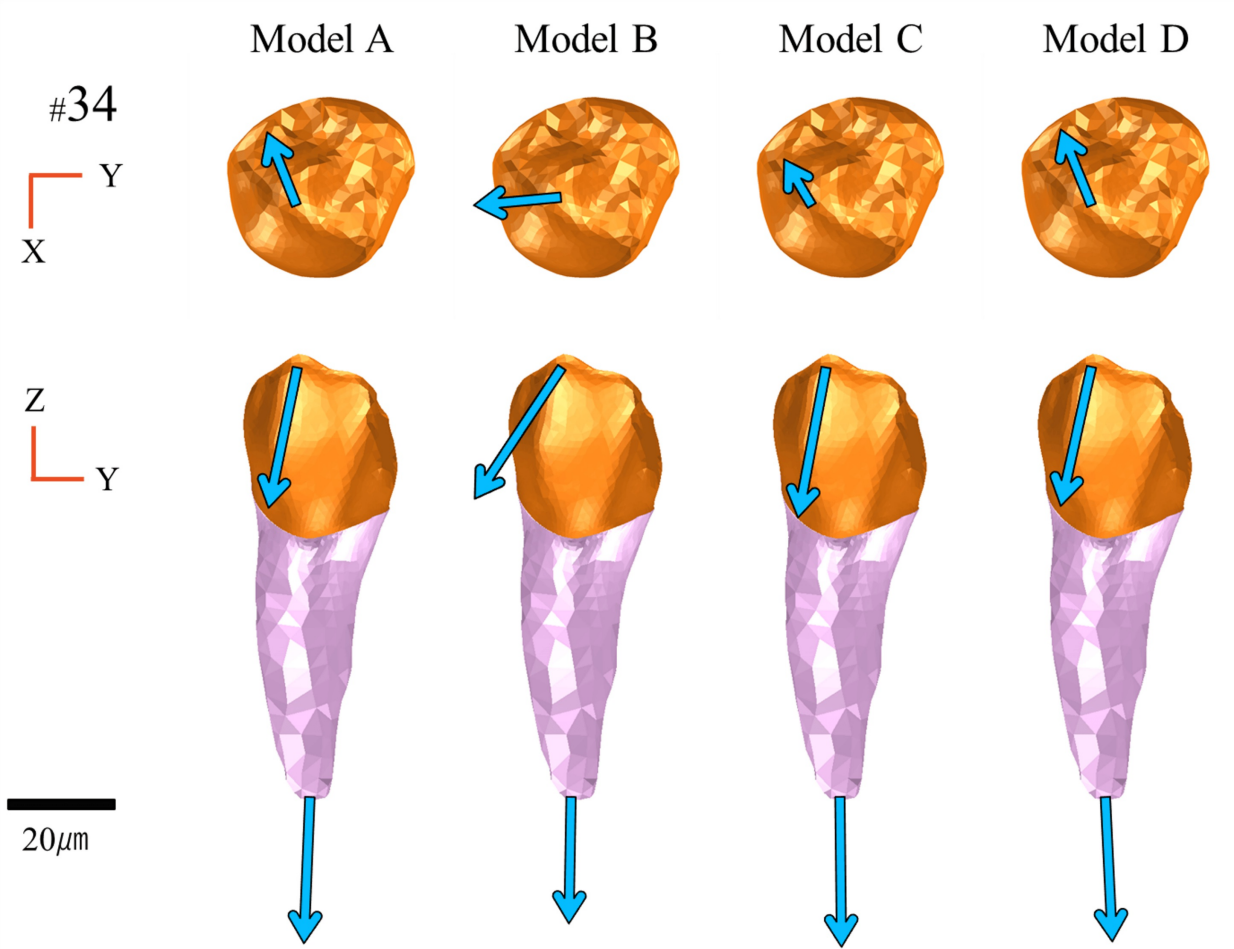
Displacement of abutment teeth (#34) Analysis models. Model A: basic model without implant support. Model B: implant placed at #45. Model C: implant placed at #46. Model D: implant placed at #47. The top and bottom rows show the occlusal and buccal views, respectively

**Figure 13:**
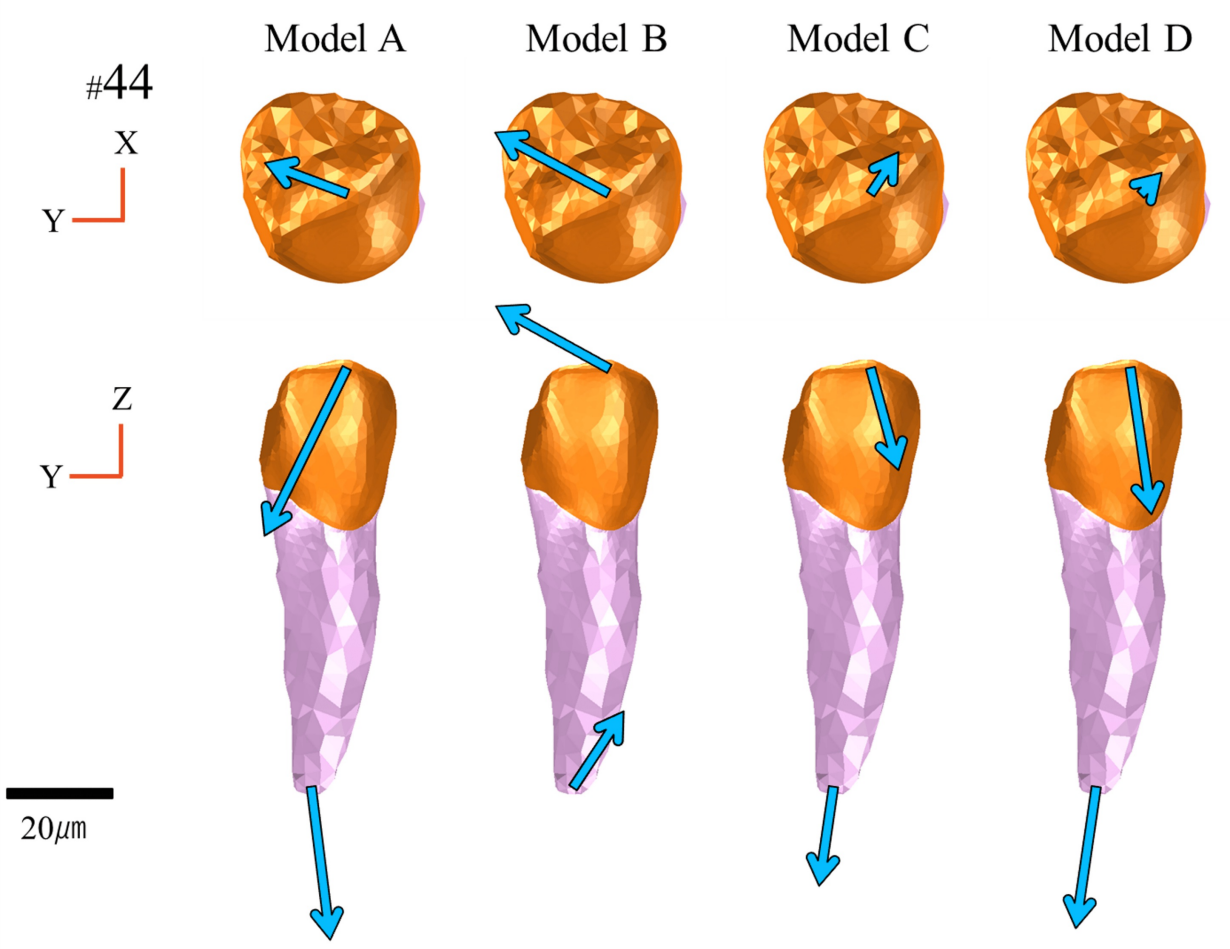
Displacement of abutment teeth (#44) Analysis models. Model A: basic model without implant support. Model B: implant placed at #45. Model C: implant placed at #46. Model D: implant placed at #47. The top and bottom rows show the occlusal and buccal views, respectively

Figures 11-13 illustrate the displacement vectors of abutment teeth #44, #34, and #37, which were equipped with direct retainers, in both the occlusal and buccal views. For tooth #37, no marked differences were observed in the displacement vector direction or magnitude in either the occlusal or buccal views among the models (Figure 11).

For tooth #34, the occlusal view exhibited reduced displacement in Model C and no marked difference in Models B and D compared with Model A. Notably, in Model B, the vector was directed buccally, a distinct orientation compared with the other models. From the buccal view, Models A, C, and D exhibited minimal differences, whereas Model B displayed mesially directed displacement with increased magnitude at the crown (Figure 12).

For tooth #44, the occlusal view revealed displacement vectors directed distolingually in Models A and B, whereas in Models C and D, they were directed mesiolingually, with magnitudes varying across models. Displacement was greater in Model B, whereas it was relatively lower in Models C and D than in Model A. From the buccal view, the vectors in Models C and D were oriented closer to the long axis of the tooth, and tended to rotate posteriorly in Models A and B (Figure 13).

Stress distribution of the residual ridge mucosa

**Figure 14:**
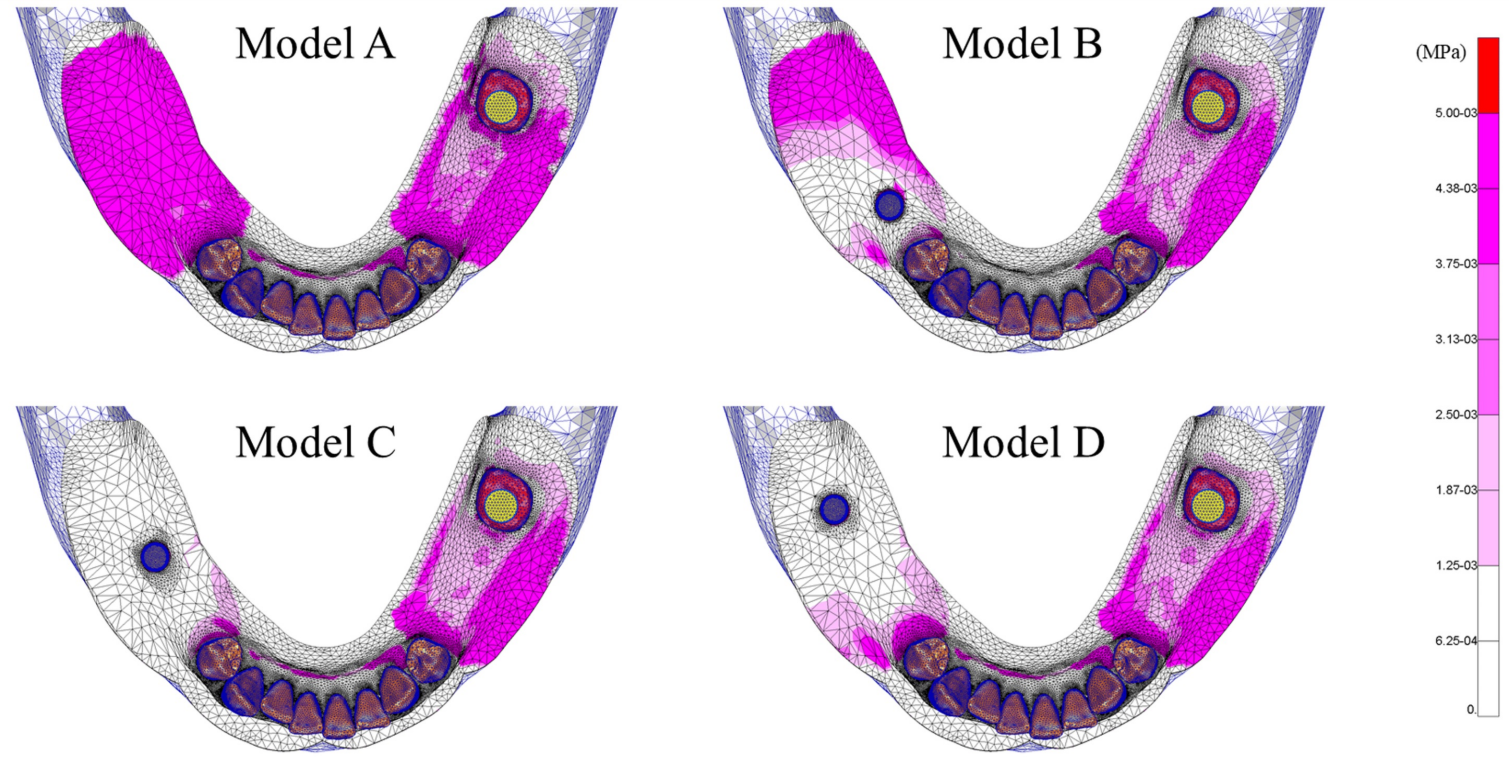
Stress distribution of the residual ridge mucosa (von Mises equivalent stress) Analysis models. Model A: basic model without implant support. Model B: implant placed at #45. Model C: implant placed at #46. Model D: implant placed at #47

Figure 14 illustrates the stress distribution of the residual ridge mucosa. Compared to Model A, stress relief was observed around the implant-supported regions of the right residual ridge mucosa. Among the implant-supported models, Models C and D exhibited greater stress relief than Model B, with Model C exhibiting the most marked reduction in stress.

Stress distribution of the alveolar bone

**Figure 15:**
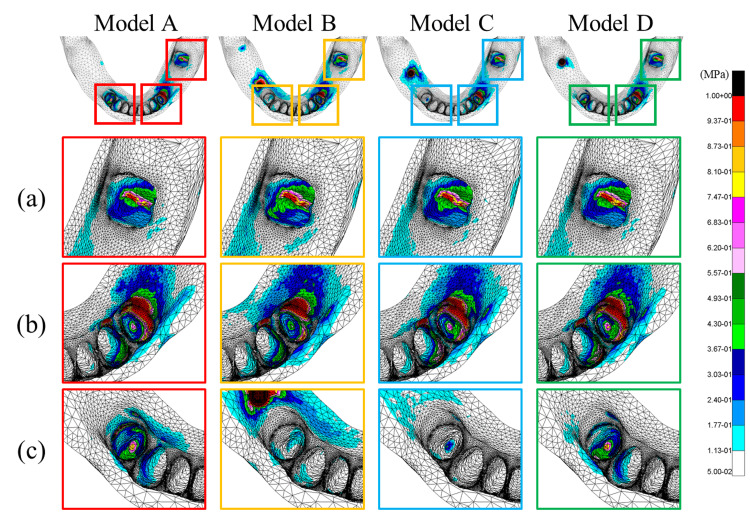
Stress distribution of the alveolar bone (von Mises equivalent stress) Analysis models. Model A: basic model without implant support. Model B: implant placed at #45. Model C: implant placed at #46. Model D: implant placed at #47. Rows (a-c) indicate the evaluated alveolar socket regions: (a) tooth #37, (b) tooth #34, and (c) tooth #44

The stress distribution in the alveolar bone surrounding each abutment tooth is illustrated in Figure 15.

Alveolar Socket Region of #37 (a)

For tooth #37, which was retained using a magnetic attachment, a slight increase in stress was observed on the mesial surface in Model B, while there was no marked difference between Model C and D.

Alveolar Socket Region of #34 (b)

For the left abutment tooth (#34), Model B demonstrated decreased stress at the base of the alveolar socket as compared to Model A. Stress tended to increase with distal implant placement.

Alveolar Socket Region of #44 (c)

For the right abutment tooth (#44), Models B and C exhibited decreased stress at the base of the alveolar socket and the surrounding bone surface. Stress levels tended to increase with distal implant placement, with no marked difference between Models D and A.

Stress distribution of the peri-implant bone

**Figure 16:**
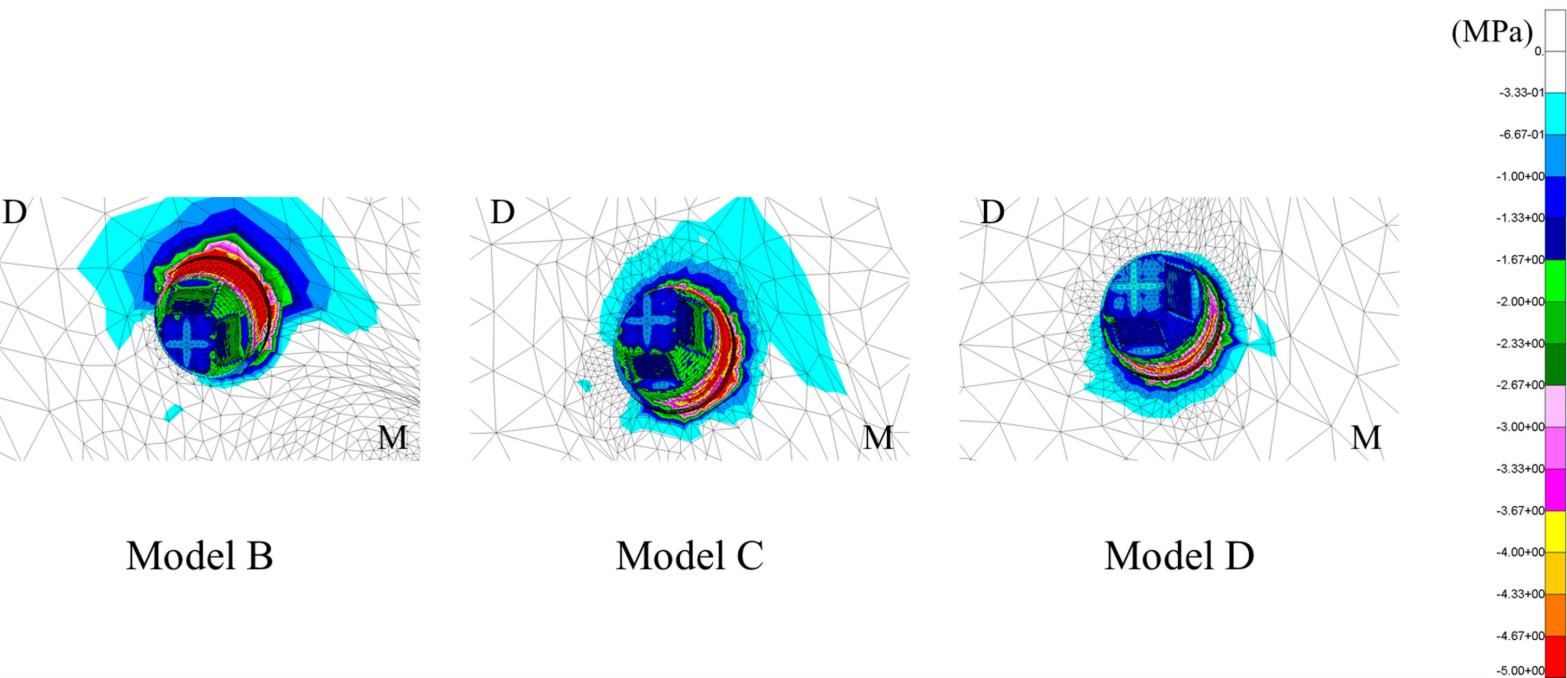
Stress distribution of the peri-implant bone (minimum principal stress) Stress distribution of the alveolar bone surrounding the implant. Analysis models. Model B: implant placed at #45. Model C: implant placed at #46. Model D: implant placed at #47 D: distal; M: mesial

The stress distribution in the peri-implant bone is illustrated in Figure 16. The minimum principal stress was used as the evaluation criterion. In all models, stress was concentrated in the cervical region of the peri-implant bone. As implant placement was increased distally, the extent of stress distribution in the peri-implant bone decreased, and the area of concentrated stress shifted mesially.

**Figure 17:**
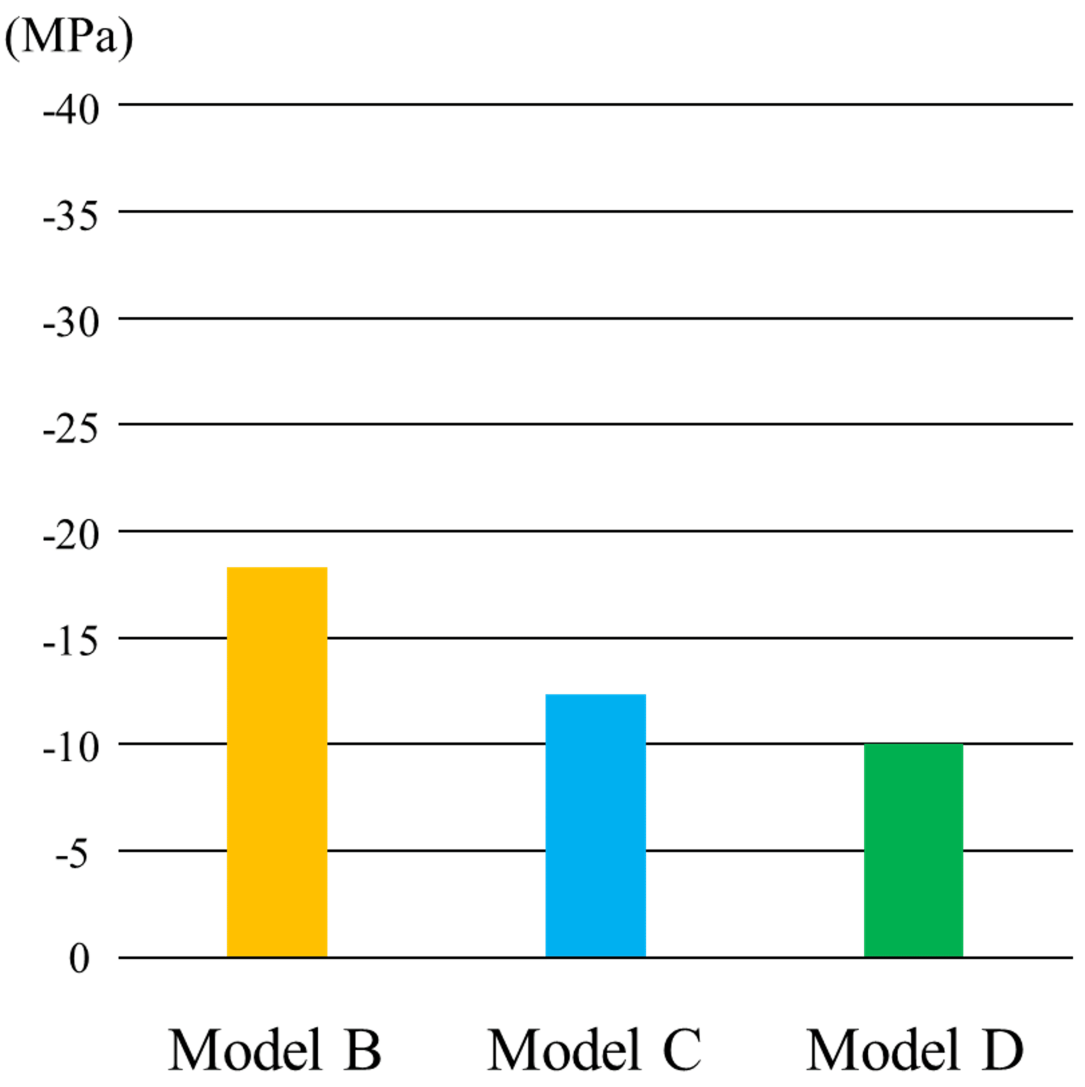
Stress values of the peri-implant bone (minimum principal stress)

Based on the minimum principal stress values, a more distal implant placement resulted in lower stress values (Figure 17).

## Discussion

Analysis model

In finite-element analysis, achieving high geometric fidelity in the computational model is essential for obtaining reliable biomechanical predictions. In the present study, high-fidelity reproduction of the model geometry was achieved by integrating shape data from the mandibular gypsum model and the skull model with digitized geometry data of the removable partial denture fabricated on the gypsum model. Furthermore, considering that the thickness of the residual ridge mucosa varies by region, site-specific mean mucosal thickness values were applied with reference to the report by Munakata et al. [10], allowing for the construction of a model that closely approximates in vivo anatomical conditions. The finite-element mesh was generated using tetrahedral and wedge elements. To improve numerical accuracy, the mesh was refined as much as possible while maintaining appropriate element aspect ratios. Collectively, these modeling procedures were expected to enhance the anatomical fidelity of the finite-element model, thereby improving the reliability of the biomechanical predictions in the present analysis.

Analysis conditions

Loading Conditions

In the oral environment, occlusal force generated during mastication is considered the primary external load acting on a removable partial denture. Therefore, in the present study, the loading conditions were designed to simulate occlusal loading on the denture. Concentrated vertical loads were applied perpendicular to the occlusal plane at six sites corresponding to the central fossae of the occlusal surfaces of teeth #35, #36, #37, #45, #46, and #47, where relatively large occlusal forces are expected. Based on the report by Manda et al. [16], a load of 50 N was applied to each site with equal distribution, resulting in a total load of 300 N.

Boundary Conditions

Boundary conditions, particularly constraint settings, can substantially influence the stress distribution and displacement behavior of the analyzed structures. Therefore, in the present study, preliminary analysis was conducted to confirm that the constraint settings did not unduly affect the results. Consequently, the region corresponding to the inferior border of the mandible, located farthest from the loading sites, was defined as fully constrained.

Contact Conditions

In stress analysis, defining contact relationships among model components is critically important. In this study, contact interactions were defined across all interfaces between the removable partial denture (including retentive components) and the remaining oral structures (including the implant healing abutment) to reproduce the contact state at these boundaries. To simulate static friction under salivary lubrication, Coulomb friction was applied, and the coefficient of friction was set to μ = 0.09, with reference to Sato et al. [17]. Collectively, these loading, boundary, and contact conditions were considered to reproduce the mechanical environment as closely as possible within the defined assumptions of the present study.

Displacement of the denture and abutment teeth

The influence of the implant placement position on the three-dimensional displacement behavior of the denture and abutment teeth was investigated. The findings in terms of vertical displacement, horizontal displacement, and overall displacement behavior are discussed below.

Vertical Displacement

Vertical displacement of the denture (Z-axis) is defined as the degree of settling, which exhibited distinct differences contingent on the implant placement site. In particular, Model C, with the implant centrally placed at site #46, exhibited the lowest degree of settling and the most favorable anteroposterior load distribution. This can be attributed to the central-support configuration, which reduced the anteroposterior rotation of the denture and facilitated even axial load distribution to the supporting tissues. In contrast, Model B exhibited greater posterior settling and associated rotational movement due to insufficient distal support. In Model D, with the implant positioned at the most distal site (#47), posterior settling was reduced, although settling was observed in the anterior region, suggesting an overall tendency for anterior rotation (Figure 9).

Regarding vertical displacement of abutment teeth, particularly at #44, Models B and C, with implants placed in close proximity to the abutment, exhibited suppressed displacement (Figure 13), suggesting that the implant may have contributed to the reduced displacement of the abutment tooth by partially supporting the occlusal load. This finding is consistent with a report by Cunha et al. [18] that implants placed closer to the abutment tooth effectively suppress vertical displacement. Moreover, in Model B, placement of the implant at site #45 generated nonphysiological extrusive forces on the abutment tooth under large distal loading, a phenomenon also reported by Murashima et al. [19].

Horizontal Displacement

Horizontal displacement (X- and Y-axes), including rotational and lateral movements, also varied depending on the implant position. Notably, in Model B, where the implant was placed at site #45, the fulcrum shifted mesially, creating a structure that was more prone to rotation at the distal-extension region, resulting in increased overall displacement of the denture (Figure 9).

Regarding the abutment teeth, horizontal displacement of site #44 was increased in Model B, with a distinctly buccally directed displacement vector (Figure 13). This increased displacement is attributed to rotational movement of the denture around the implant fulcrum, which concentrated lateral forces on the abutment.

Matsudate et al. [20] reported that in ISRPDs supported by mesially placed implants, the horizontal force components acting on both the abutment teeth and implants increase and exert a greater influence than the vertical forces. In contrast, Models C and D, with the fulcrum located more distally, exhibited reduced rotational movement with minimal horizontal displacement of the abutment teeth.

Comprehensive Displacement Behavior

The implant placement position clearly influenced the three-dimensional displacement behavior of the denture and abutment teeth. Placement at site #45 contributed to reducing the axial load on the adjacent abutment but concurrently promoted denture rotation and increased lateral displacement of the abutment. Placement at site #47 effectively mitigated distal settling but demonstrated limited efficacy in reducing abutment tooth displacement, suggesting minimal benefit for mechanical load distribution to the abutment. In contrast, implant placement at the central site (#46) provided the most favorable overall biomechanical trend under the present modeling conditions in mitigating denture settling and rotation, while minimizing abutment displacement, thereby suggesting improved mechanical stability.

Stress distribution of the residual ridge mucosa

Stress distribution of the residual ridge mucosa showed marked differences depending on the implant placement position. In Model B, due to insufficient posterior support, the denture settled distally, resulting in a broad distribution of stress across the mucosal surface. In contrast, Model C provided well-balanced support in the anteroposterior direction, leading to the lowest amount of stress to the residual ridge mucosa with effective distribution of the overall load. In Model D, although stress of the posterior mucosa was reduced, the denture exhibited anterior settling due to support from the anterior abutment teeth, causing stress to be distributed in the anterior mucosal region. Previous studies by Tun Naing et al. [21] and Memari et al. [22] likewise indicate that placing the implant at site #46 yielded the most favorable stress distribution of the residual ridge mucosa.

Stress distribution of the alveolar bone surrounding the abutment teeth

Distinct differences in stress distribution of the alveolar bone surrounding the abutment tooth (#44) were dependent on the implant placement position. In Models B and C, the implant was located closer to the abutment tooth, thereby allowing occlusal forces to be partially dispersed through the implant, resulting in reduced stress of the surrounding alveolar bone. In contrast, Model D did not achieve load sharing with the abutment tooth because the implant was placed more distally and the stress levels remained comparable to those of Model A. Kihara et al. [23] also reported that with Kennedy Class II cases, implant placement in close proximity to the abutment tooth reduced stress of the alveolar bone, which is consistent with the findings of the present study. In Model B, rotation of the denture on the implant at site #45 shifted part of the load from the vertical to lateral direction [20], resulting in slightly higher local stress of the alveolar bone surface as compared to Model C. Conversely, no substantial differences were observed in the stress distribution of the abutment teeth on the nonimplant side (sites #34 and #37) among the models. These findings suggest that placement of the implant closer to the abutment tooth can reduce stress of the surrounding alveolar bone.

Stress distribution of the peri-implant bone

In all models, minimum principal stress was concentrated to the cervical region of the peri-implant bone. Furthermore, as placement of the distal implant was more distal, the extent of stress distribution of the peri-implant bone had decreased, as did the absolute stress values, suggesting that placement of the implants more distally helped to distribute occlusal loads more effectively, thereby alleviating concentrated stress of the peri-implant bone induced by occlusal forces. Finite-element analysis conducted by Ohyama et al. [24] also demonstrated that distal implant placement reduced the stress concentration, consistent with the findings of this study.

Additionally, according to a report by Sugiura et al. [25], the stress values of the peri-implant bone in this study were all within physiological limits, which may indicate a low mechanical risk range under the present assumptions; however, clinical risks (e.g., bone resorption or complications) cannot be determined from this single static numerical model without experimental/clinical validation.

Limitations and future directions

The three-dimensional FEM was utilized to elucidate the biomechanical impact of implant placement on denture displacement, abutment tooth behavior, and stress distribution within the residual ridge mucosa and peri-implant bone. However, the analysis was conducted under static loading conditions, which did not account for the dynamic and lateral components of masticatory forces. Additionally, the model did not reflect individual biological variations, such as bone density and mucosal thickness, highlighting the need for patient-specific modeling in future studies. Furthermore, this model did not include time-dependent biological responses, such as bone remodeling, tissue adaptation, or healing; thus, long-term clinical outcomes cannot be inferred from this single static numerical analysis. In addition, the present analysis was performed using a single mandibular morphology and a specific prosthesis and implant configuration under defined modeling assumptions, which may limit the generalizability of the findings. Hence, future studies should explore the effects of the type of attachment system, the number of implants, and the overall denture design on stress distribution and denture stability to improve clinical outcomes. Because the numerical model was not validated against experimental or clinical measurements, the findings should be interpreted cautiously as biomechanical trends under the defined conditions.

## Conclusions

This finite-element study evaluated the mechanical effects of different implant placement positions with a Kennedy Class II removable partial denture. Among the three positions examined, implant placement in the first molar region (site #46) most effectively minimized denture base displacement and reduced stress on the supporting mucosa and the adjacent abutment tooth (site #44). Although minor variations in stress distribution were observed in the contralateral abutments on the nonimplant side (sites #34 and #37), these differences were negligible compared to the right side and did not indicate a substantial mechanical impact. Unlike previous studies that focused primarily on peri-implant or adjacent structures, the present study comprehensively assessed the biomechanical behavior of the entire prosthesis using a patient-derived mandibular model. These findings suggest that the implant placement position has a clear influence on the mechanical behavior of the prosthetic system, and that central placement at #46 may offer the most favorable biomechanical trend under the present modeling conditions in unilateral distal-extension ISRPDs.
